# DNA methylation profiles reveal sex-specific associations between gestational exposure to ambient air pollution and placenta cell-type composition in the PRISM cohort study

**DOI:** 10.1186/s13148-023-01601-x

**Published:** 2023-12-01

**Authors:** Hachem Saddiki, Xueying Zhang, Elena Colicino, Ander Wilson, Itai Kloog, Robert O. Wright, Rosalind J. Wright, Corina Lesseur

**Affiliations:** 1 Department of Environmental Medicine and Public Health, Icahn School of Medicine at Mount Sinai, 1 Gustave L. Levy Place, Box 1057, New York, NY 10029 USA; 2 Department of Pediatrics, Icahn School of Medicine at Mount Sinai, The Kravis Children’s Hospital, New York, NY USA; 3 Institute of Exposomic Research, Icahn School of Medicine at Mount Sinai, New York, NY USA; 4 Department of Statistics, Colorado State University, Fort Collins, CO USA; 5 Department of Geography and Environmental Development, Ben-Gurion University of the Negev, Beersheba, Israel

**Keywords:** Compositional regression, Fine particulate matter, Placenta, Cell types, DNA methylation

## Abstract

**Background:**

Gestational exposure to ambient air pollution has been associated with adverse health outcomes for mothers and newborns. The placenta is a central regulator of the in utero environment that orchestrates development and postnatal life via fetal programming. Ambient air pollution contaminants can reach the placenta and have been shown to alter bulk placental tissue DNA methylation patterns. Yet the effect of air pollution on placental cell-type composition has not been examined. We aimed to investigate whether the exposure to ambient air pollution during gestation is associated with placental cell types inferred from DNA methylation profiles.

**Methods:**

We leveraged data from 226 mother–infant pairs in the Programming of Intergenerational Stress Mechanisms (PRISM) longitudinal cohort in the Northeastern US. Daily concentrations of fine particulate matter (PM_2.5_) at 1 km spatial resolution were estimated from a spatiotemporal model developed with satellite data and linked to womens’ addresses during pregnancy and infants’ date of birth. The proportions of six cell types [syncytiotrophoblasts, trophoblasts, stromal, endothelial, Hofbauer and nucleated red blood cells (nRBCs)] were derived from placental tissue 450K DNA methylation array. We applied compositional regression to examine overall changes in placenta cell-type composition related to PM_2.5_ average by pregnancy trimester. We also investigated the association between PM_2.5_ and individual cell types using beta regression. All analyses were performed in the overall sample and stratified by infant sex adjusted for covariates.

**Results:**

In male infants, first trimester (T1) PM_2.5_ was associated with changes in placental cell composition (*p* = 0.03), driven by a decrease [per one PM_2.5_ interquartile range (IQR)] of 0.037 in the syncytiotrophoblasts proportion (95% confidence interval (CI) [− 0.066, − 0.012]), accompanied by an increase in trophoblasts of 0.033 (95% CI: [0.009, 0.064]). In females, second and third trimester PM_2.5_ were associated with overall changes in placental cell-type composition (T2: *p* = 0.040; T3: *p* = 0.049), with a decrease in the nRBC proportion. Individual cell-type analysis with beta regression showed similar results with an additional association found for third trimester PM_2.5_ and stromal cells in females (decrease of 0.054, *p* = 0.024).

**Conclusion:**

Gestational exposure to air pollution was associated with placenta cell composition. Further research is needed to corroborate these findings and evaluate their role in PM_2.5_-related impact in the placenta and consequent fetal programming.

**Supplementary Information:**

The online version contains supplementary material available at 10.1186/s13148-023-01601-x.

## Background

Gestational exposure to fine ambient particulate matter (PM_2.5_) has been linked to adverse pregnancy outcomes, particularly increased risk of preterm birth and infant low birth weight [1, 2]. The placenta has been suggested as a target organ of air pollution exposures during gestation with potential impact on adverse pregnancy outcomes [3, 4]. Evidence from several studies suggest that air pollution can affect fetal growth via *in utero* oxidative stress, systemic inflammation and impaired vascularization [3, 5, 6]. Placental molecular marks including DNA methylation have been proposed as a mechanism linking PM_2.5_ to fetal growth [7–10]. Importantly, evidence from multiple studies suggest that the effects of exposure to air pollution can differ based on timing of exposure, with certain gestational weeks showing stronger associations than other time periods in gestation [8, 11, 12]. Additionally, fetal sex plays a crucial role, as evidenced by studies documenting sex-specific associations between air pollution and anthropometric and developmental outcomes in offspring [13–15]. Similarly, sex differences have been reported in placental function, gene expression and DNA methylation marks [7, 16–20]. To our knowledge, no prior study has investigated the potential impact of gestational exposure to PM_2.5_ on placental cell-type composition exploring sex-related differences in these associations.

A mature placenta consists of different cell types with distinct function and epigenetic patterns including trophoblasts, syncytiotrophoblasts, Hofbauer cells (placental macrophages), stromal, endothelial and nucleated red blood cells (nRBCs) [21]. Dysregulation of specific cell types likely plays a role in pregnancy outcomes. A recent study that used cell-type deconvolution of bulk RNA-seq placenta datasets reported that preeclampsia was associated with an increase in extra-villous trophoblasts and lower mesenchymal cells [22]. Yet, there is little knowledge on the role of placental cell types or changes in cell-type composition in the development of specific pregnancy and placenta outcomes. Exposure to environmental pollutants has been associated with the development of placental abnormalities, although the molecular underpinnings remain poorly delineated. While cell-type composition is likely important in this context, limited information exists on the effect of pollutants, including PM_2.5,_ on placental cell-type composition. A recent study compiled a DNA methylation reference for placental cells that can be leveraged for reference-based deconvolution of bulk tissue samples in epidemiological studies [23]. More recently, another study that evaluated the reliability of this method showed that reference-based deconvolution of placental samples reflects previous knowledge from histology studies of gestation-induced changes in placental cell-type proportions [24]. To date, epidemiological studies examining the effects of PM_2.5_ on the placenta largely consider epigenetic profiles derived from bulk tissue samples composed of a heterogeneous mixture of cells [9, 25].

Herein, we derived the placental cell-type proportion estimates from DNA methylation data and then examined associations between prenatal PM_2.5_ exposure and placenta cell-type composition in participants from the urban PRogramming of Intergenerational Stress Mechanisms (PRISM) study, a longitudinal pregnancy cohort established in the Northeastern US. We also examined sex-specific effects and time-varying associations between PM_2.5_ exposure and placental cell types by leveraging the longitudinal measurements of air pollution in this cohort. Our study aims to fill the knowledge gap regarding the influence of gestational exposure to PM_2.5_ on placental cell-type composition, which is crucial for understanding the molecular mechanisms underlying the effects of environmental pollutants on the placenta.

## Methods

### Study design and participants

We leveraged data from the PRISM study, a prospective pregnancy cohort designed to examine the effects of prenatal and early life psychosocial and other environmental exposures on child developmental outcomes. Beginning in 2011, pregnant women receiving prenatal care from the Beth Israel Deaconess Medical Center and East Boston Neighborhood Health Center in Boston, Massachusetts as well as Mount Sinai Hospital in New York City (NYC), New York, were recruited in mid- to late-pregnancy. Women were considered eligible if they were English or Spanish speaking, 18 years or older and pregnant with a singleton. Exclusion criteria included maternal intake of ≥ 7 alcoholic drinks per week prior to pregnancy, any alcohol intake after pregnancy recognition, HIV + status, or delivery of an infant born with congenital abnormalities that would impede ongoing participation. At the time of these analyses, 1109 mother–child dyads, including 399 from Boston (enrolled March 2011–October 2013) and 710 from NYC (enrolled April 2013–February 2020), were enrolled prenatally, with continued active follow-up. Supplemental funding was obtained to collect placenta samples at birth for DNA isolation and DNA methylation analyses on a subset leveraged herein. Procedures were approved by the relevant institutions’ human studies committees, and written informed consent was obtained from women prior to study participation in their preferred language.

### ***PM***_***2.5***_*** exposure assessment***

As previously detailed [15], geocoding of residential address history for all pregnant women was conducted by a Geographic Information Systems specialist using Arc Geographic Information Systems (ArcGIS). Daily concentrations of ambient PM_2.5_ were estimated for each participant using hybrid models that combined satellite-retrieved products, meteorology data, reanalysis atmospheric models and land use variables to make predictions at a1 km × 1 km spatial resolution (Just et al., 2020). The modeled daily PM_2.5_ levels were validated through regressing the PM_2.5_ measured using filter-based monitors (obtained by US EPA Air Quality System and Interagency Monitoring of Protected Visual Environments Network), resulting in a robust out of sample tenfold cross-validation (*R*^2^ = 0.87). The model demonstrates excellent predictions of withheld observations (overall RMSE of 2.10 μg/m^3^ and RMSE of 3.11 μg/m^3^ in our spatial cross-validation). Records of relocation were documented, and geocoding was done for all address histories if a participant moved during pregnancy and daily PM_2.5_ estimate for participants that moved was based on location and time at each address. We calculated weekly average exposure and trimester-average exposure by taking the arithmetic mean of daily PM_2.5_ concentrations. The weekly average and trimester-average PM_2.5_ concentrations were used to examine associations between air pollution and placental cell types in compositional [26] and beta regression analyses.

### Placenta DNA methylation and cell-type deconvolution

Immediately after birth, placental tissue samples (~ 1 cm^3^) were taken from the fetal side of the placenta approximately 4 cm from the umbilical cord insertion in four quadrants and below (~ 1–1.5 cm) the chorionic membranes avoiding large vessels as per a published protocol [27]. To avoid contamination, visible decidua and membranes were removed, and samples were rinsed in a cold PBS bath. Each tissue sample was cut in smaller pieces (~ 0.1 cm^3^) and placed into RNAlater™ Stabilization Reagent (Qiagen, Germantown, MD) and placed at − 4 °C for ≤ 24 h, then excess RNAlater was removed and samples were stored at − 80 °C until DNA isolation. DNA was isolated using the Gentra Puregene kit (Qiagen, Germantown, MD) following manufacturer’s protocols and quantified in the Implen Nanophotometer Pearl (Westlake Village, CA) [19]. 500 ng DNA were bisulfite converted using the EZ DNA Methylation-Gold™ Kit (Zymo Research, Orange, CA). DNA methylation was measured with the Infinium HumanMethylation450 array (Illumina, San Diego, CA) on consecutive runs at the Partners HealthCare Translational Genomics Core Labs. To avoid batch effects, samples were arranged on chips with a stratified randomization followed by checks for balance on specific characteristics (birthweight z-score, gestational age, sex and study site). The Infinium HumanMethylation450 array (Illumina) measures DNA methylation at 485,577 sites located throughout the genome. Methylation values at each CpG site are computed as the methylated intensity divided by the sum of the methylated and unmethylated intensities measured at fluorescence site-specific probes. Methylation values ranged from zero (fully unmethylated) to one (fully methylated). The processing of raw methylation data (.idat files) was performed as previously described [19] using the R package ewastools [28]. Quality control steps included: outlier detection with principal components analysis (PCA), assessment of sample swapping or contamination using 65 SNP probes, checking for infant sex mismatches with X and Y chromosome probes, dye bias correction performed with RELIC [29], removing observations with detection *p*-values < 0.05 and exclusion of samples that did not follow the expected bimodal distribution. We excluded probes in sex chromosomes (*n* = 11,648), probes known to be cross-reactive (*n* = 28,298) or within 10 bp of a SNP (MAF ≥ 0.05 in American admixed population, *n* = 30,067) and probes with missing values in ≥ 10% of samples (*n* = 426). Batch effects were evaluated using PCA, and batch effects of plate were removed using ComBat [30]. After quality control, 229 samples and 415,073 probes were used for analyses.

Reference-based cell-type deconvolution was performed using the planet R package [23] and the constrained projection method (Houseman et al. 2012) to estimate the proportions of six cell types: syncytiotrophoblasts, trophoblasts, stromal, endothelial, Hofbauer and nucleated red blood cells (RBC). Finally, since compositional regression does not allow cell-type proportions including zeros when using log-ratio transformations, we imputed the rounded zeros in the estimated cell-type proportions using the “robCompositions” package in R [31, 32]. The method used to impute the zeros employs partial least square regression with Q-mode clustering as described in Chen et al. [33]. The method replaces zeros with small numbers akin to detection limits of the cell proportions while still conserving the geometry of the compositional space; this hardly affects the cell proportion estimates, yet still allows the application of log-ratio transformation for the compositional regression [33].

### Covariates

Information on race/ethnicity, age and education were collected using standardized questionnaires administered during an in-person study visit conducted at enrollment. Gestational age at birth was estimated from (1) last menstrual period and (2) obstetrical estimates; if the discrepancy between the two sources was greater than two weeks, obstetrical estimates were used. Information on diagnosis with gestation-related health conditions (eclampsia, preeclampsia) was abstracted from the electronic medical record.

### Statistical analyses

The analytic sample in this study is composed of 226 mother–child pairs with complete data on placental DNA methylation, PM_2.5_ exposure and relevant covariates. Descriptive statistics were conducted for study exposure, outcomes and covariates. We compared study variables of mother–infant dyads among male and female infants using Student’s t test for continuous variables and chi-square for categorical variables, respectively. We used compositional regression to examine the association between trimester-average PM_2.5_ concentrations and the proportions of six placenta cell types [26, 34]. The linear regression model for compositions is a close analog to the classical multivariate linear regression model; the main difference is that the compositional model simultaneously analyzes the six cell-type proportions as a single multivariate outcome, through an isometric log-ratio transformation. Previous research has demonstrated the potential differential effects of air pollution by fetal sex and trimester of exposure [11, 35–37]. Therefore, we fitted an “overall” model that included all participants and separate models stratifying by child sex. We included all three trimester-average PM_2.5_ exposures as predictors in each model, which has been shown to reduce bias compared to models that analyze exposure in each trimester separately. IQR standardization was applied to each trimester-average PM_2.5_ exposure level. We report 95% bootstrapped confidence intervals and the p-values of bootstrap-corrected tests based on 1000 bootstrap samples to evaluate the association between each trimester-average PM_2.5_ exposure and the expected change in cell composition. Bootstrapped confidence intervals have been previously used in the context of compositional analyses [38]. All results are reported in terms of one IQR increase in exposure, and all analyses were performed using R with the package “compositions” [34].

Furthermore, we perform a secondary analysis to further corroborate the results from our compositional regression models. We use beta regression models to investigate the association between PM_2.5_ exposures and each individual cell-type proportion separately; beta regression was designed to model the outcome as a proportion, but it is not able to jointly model multiple proportions that sum up to one. For each cell type, we report the estimated regression coefficients associated with PM_2.5_ exposure during each trimester as well as 95% confidence intervals (CI) and *p*-values. Beta regression analyses were performed using R with the package “betareg” [39]. We also performed sensitivity analyses by excluding participants with severe pregnancy complications (preeclampsia or eclampsia) to evaluate the robustness of our analyses. All models were adjusted for infant sex (overall analyses), maternal age at birth and race/ethnicity, gestational age and season of birth, each model included PM_2.5_ exposure for all three trimesters to minimize bias. Other covariates like maternal education and smoking were also considered, but these had little impact on the results and were not included the final models.

## Results

**Fig. 1:**
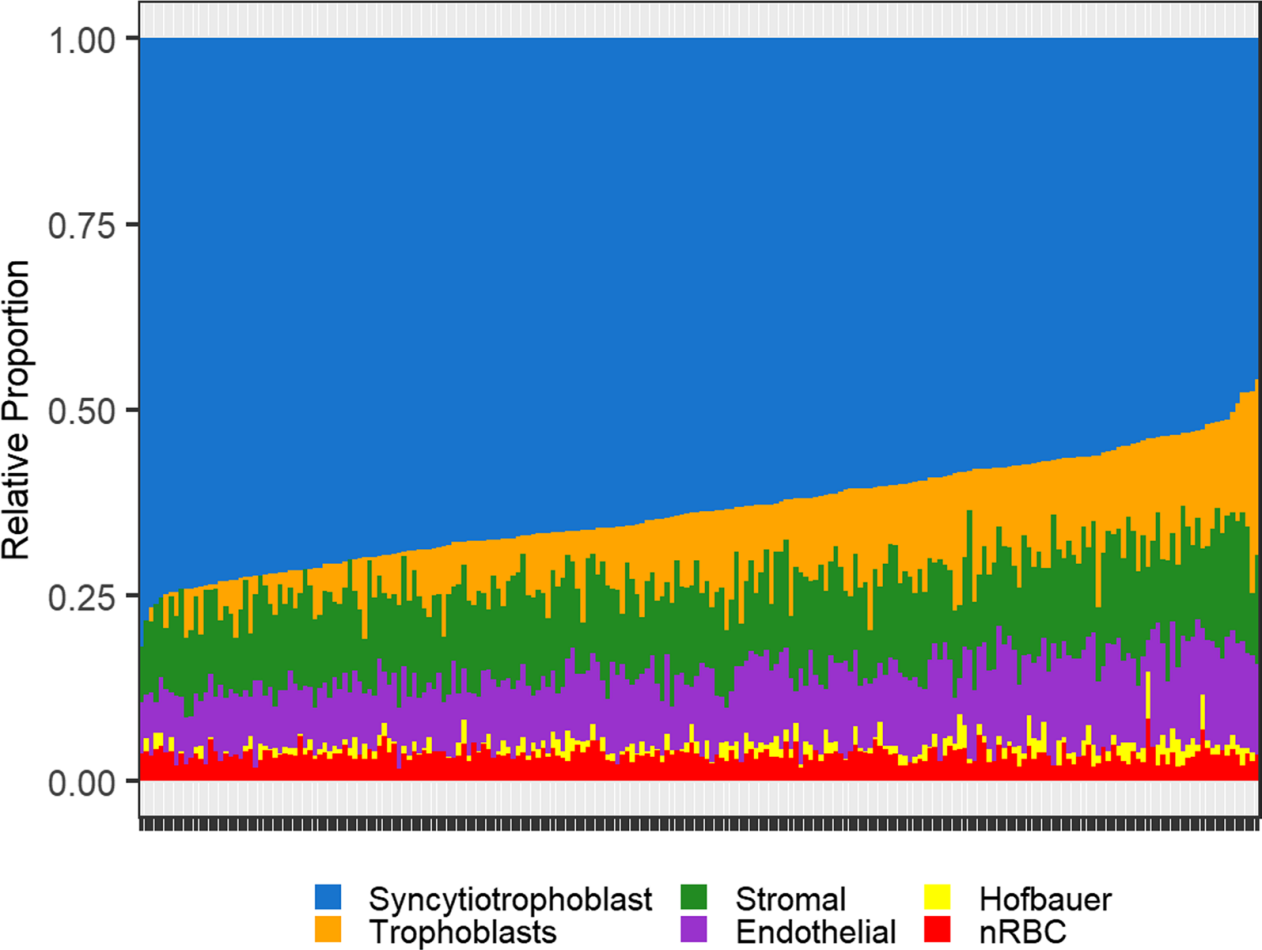
Stacked bar plots showing the relative proportions of the estimated placenta cell types, each participant is a bar in the x-axis (N = 226). The samples were sorted in decreasing order of syncytiotrophoblasts proportion

The clinical and demographic characteristics of the analytic sample are shown in Table 1, both for the overall sample and stratified by sex. Among the newborns included in this study, 101 (44.7%) were females and the mean gestational age was 39 weeks [standard deviation (SD) 1.5 weeks]. Maternal race/ethnicity, education and age at birth did not differ significantly between female and male infants. Among the analytic sample, 17 mothers were diagnosed with preeclampsia or eclampsia during pregnancy. The average PM_2.5_ exposure was slightly higher in the third trimester than in the first two trimesters. Detailed information on the median and IQR of PM_2.5_ exposures stratified by trimester can be found in Additional file 1: Table S1. Figure 1 shows the distribution of the six distinct cell types from placenta tissue DNA methylation using the reference-based deconvolution method. The syncytiotrophoblast was the predominant cell type, accounting for an average of 63% across samples, followed by the stromal (mean 12.7%) and endothelial cells (mean 9.9%). Nucleated RBCs (nRBCs) and placental macrophages (Hofbauer cells) were the least abundant, averaging 3.6% and 1.2%, respectively. Notably, the proportions of three cell types (syncytiotrophoblast, trophoblasts and Hofbauer) differed significantly by child sex, as shown in Table 1.

**Table 1:** Characteristics of the study population, PM_2.5_ exposure and placenta cell types

	All (*N* = 226)	Female (*N* = 101)	Male (*N* = 125)	*P*-value
Gestational age (wks.), mean (SD)	38.96 (1.50)	39.04 (1.50)	38.90 (1.50)	0.32
*Maternal race/ethnicity, N (%)*				
White	72 (31.86%)	30 (29.70%)	42 (33.60%)	0.59
Black	91 (40.27%)	38 (37.62%)	53 (42.40%)	0.47
Hispanic	46 (20.35%)	25 (24.75%)	21 (16.80%)	0.47
Other	14 (6.19%)	8 (7.92%)	6 (4.80%)	0.54
*Maternal education, N (%)*				
High school or less, N (%)	61 (26.99%)	33 (32.67%)	28 (22.40%)	0.09
Maternal age at birth, mean (SD)	30.57 (5.59)	30.68 (5.87)	30.48 (5.38)	0.59
*Pregnancy complications, N (%)*				
Preeclampsia	15 (6.36%)	5 (4.95%)	10 (8%)	0.63
Eclampsia	2 (0.88%)	0 (0.00%)	2 (1.60%)	1.00
*Season of birth, N (%)*				
Spring (March to May)	59 (26.11%)	25 (24.75%)	34 (27.20%)	0.87
Summer (June – August)	54 (23.89%)	26 (25.74%)	28 (22.40%)	0.91
Fall (September – November)	52 (23.01%)	24 (23.76%)	28 (22.40%)	0.93
Winter (December – February)	61 (26.99%)	26 (25.74%)	35 (28%)	0.98
*PM*_*2.5*_* exposures, mean (SD)*				
Pregnancy	8.33 (0.97)	8.42 (0.99)	8.25 (0.95)	0.19
First trimester	8.25 (1.36)	8.40 (1.45)	8.12 (1.28)	0.14
Second trimester	8.24 (1.34)	8.28 (1.43)	8.20 (1.26)	0.65
Third trimester	8.56 (1.77)	8.66 (1.69)	8.47 (1.83)	0.42
*Cell-type proportions, mean (SD)*				
Syncytiotrophoblast	0.636 (0.069)	0.623 (0.074)	0.644 (0.064)	0.023
Trophoblasts	0.091 (0.049)	0.099 (0.052)	0.084 (0.046)	0.020
Stromal	0.127 (0.026)	0.129 (0.026)	0.125 (0.026)	0.244
Endothelial	0.099 (0.027)	0.102 (0.025)	0.096 (0.028)	0.072
Hofbauer	0.012 (0.011)	0.010 (0.009)	0.014 (0.011)	0.003
nRBC	0.036 (0.010)	0.036 (0.009)	0.036 (0.010)	0.753

In compositional analysis (Table 2), we did not detect significant associations between PM_2.5_ exposure and placental cell-type composition in the overall model, which included both male and female infants. However, when examining sex-stratified models, we found significant associations between second and third trimester PM_2.5_ exposures and overall changes in placental cell-type composition among female infants. Specifically, one IQR increase in third trimester PM_2.5_ among female infants was significantly associated with a decrease of 0.003 (95% CI: [− 0.005, − 0.001%]) in nRBCs proportion. We also detected a significant change (*p* = 0.040) in overall cell-type composition associated with increased PM_2.5_ in second trimester among female infants; however, yet we did not identify a particular cell type is driving this association. In contrast, among males, first trimester PM_2.5_ was significantly associated with changes in overall cell-type composition (*p* = 0.032), particularly in the syncytiotrophoblast and trophoblast cell proportions. For males, an IQR increase in first trimester PM_2.5_ was associated with a decrease of 0.037 (95% CI: [− 0.066, − 0.012%]) in the syncytiotrophoblast proportion and an increase of 0.033 (95% CI: [0.009, 0.064]) in the trophoblast proportion. Meanwhile, the proportion of stromal cells (0.001, 95%CI: [− 0.009, 0.009]), endothelial cells (0.003, 95% CI: [− 0.006, 0.011]), Hofbauer cells (0.002. 95% CI: [− 0.002, 0.011]) and nRBC (− 0.001, 95%CI: [− 0.003, 0.002]) did not exhibit significant changes.

**Table 2:** Composition analyses between trimester-average PM_2.5_ and placental cell types

Effect of PM_2.5_	Syncytiotrophoblasts	Trophoblasts	Stromal	Endothelial	Hofbauer	nRBC	*p*-value*
*Overall*							
T1	− 0.013 (− 0.034, 0.007)	0.015 (− 0.002, 0.035)	− 0.004 (− 0.010, 0.002)	0.002 (− 0.003, 0.007)	0 (− 0.002, 0.002)	0 (− 0.002, 0.002)	0.115
T2	0.005 (− 0.007, 0.017)	− 0.002 (− 0.011 0.007)	− 0.003 (− 0.008, 0.002)	− 0.003 (− 0.007, 0.002)	0.001 (0, 0.003)	0.001 (0, 0.003)	0.067
T3	0.002 (− 0.015, 0.016)	− 0.005 (− 0.018, 0.008)	0.004 (− 0.002, 0.009)	0 (− 0.005, 0.006)	0 (− 0.001, 0.001)	0 (− 0.003, 0.001)	0.162
*Female*							
T1	0.005 (− 0.027, 0.036)	0.003 (− 0.020, 0.032)	− 0.007 (− 0.017, 0.002)	0 (− 0.008, 0.008)	− 0.001 (− 0.001, 0.001)	0 (− 0.003, 0.003)	0.160
T2	0.017 (− 0.001, 0.033)	− 0.010 (− 0.023, 0.004)	− 0.006 (− 0.012, 0.002)	− 0.003 (− 0.009, 0.003)	0 (− 0.001, 0.002)	0.001 (− 0.001, 0.004)	**0.040**
T3	− 0.002 (− 0.030, 0.019)	− 0.003 (− 0.024, 0.021)	0.004 (− 0.004, 0.013)	0.004 (− 0.003, 0.011)	0 (− 0.001, 0.002)	** − 0.003 (− 0.005, − 0.001)**	**0.049**
*Males*							
T1	** − 0.037** (**− 0.066, − 0.012)**	**0.033 (0.009, 0.064)**	0.001 (− 0.009, 0.009)	0.003 (− 0.006, 0.011)	0.002 (− 0.002, 0.011)	− 0.001 (− 0.003, 0.002)	**0.033**
T2	− 0.009 (− 0.027, 0.009)	0.006 (− 0.007, 0.020)	0.001 (− 0.007, 0.008)	− 0.002 (− 0.009, 0.005)	0.003 (− 0.001, 0.011)	0.001 (− 0.001, 0.003)	0.117
T3	0.010 (− 0.011, 0.028)	− 0.010 (− 0.023, 0.007)	0.002 (− 0.005, 0.010)	− 0.002 (− 0.009, 0.005)	− 0.001 (− 0.003, 0.002)	0.001 (− 0.001, 0.004)	0.131

^*^Overall effects on cell-type composition *p*-value calculated based on the median F-statistic across 1000 bootstrap samples. In parenthesis are the 95% bootstrap confidence intervals. Significant results are highlighted in bold. T1: 1st trimester, T2: 2nd trimester, T3: 3rd trimester, nRBC: nucleated red blood cells

We obtained similar results in the individual cell-type analyses using Beta regression models (Table 3). Among male infants, first trimester PM_2.5_ exposure was significantly associated with an increase in the proportion of trophoblasts (log-odds of 0.273, *p* = 0.005) accompanied by a non-significant decrease in the syncytiotrophoblast fraction. In females, third trimester PM_2.5_ exposure was associated with a decrease in log-odds of 0.088 (*p* = 0.015) in nRBCs, and we also observed that second trimester PM_2.5_ exposure was significantly associated with a decrease in log-odds of 0.054 (*p* = 0.024) in stromal cells.

**Table 3:** Associations between PM_2.5_ exposure and individual placental types estimated using Beta regressions

Effect of PM_2.5_	Syncytiotrophoblasts	Trophoblasts	Stromal	Endothelial	Hofbauer	nRBC
*Overall*						
T1	− 0.019 (− 0.080, 0.041)	0.128 (− 0.008, 0.264)	− 0.023 (− 0.071, 0.025)	0.032 (− 0.029, 0.092)	− 0.024 (− 0.223, 0.175)	0.001 (− 0.055, 0.056)
T2	0.018 (− 0.026, 0.061)	− 0.02 (− 0.119, 0.079)	− 0.027 (− 0.061, 0.007)	− 0.029 (− 0.073, 0.015)	0.105 (− 0.038, 0.248)	0.027 (− 0.013, 0.067)
T3	− 0.010 (− 0.062, 0.042)	− 0.033 (− 0.153, 0.088)	0.026 (− 0.015, 0.066)	0 (− 0.052, 0.052)	− 0.042 (− 0.212, 0.128)	− 0.023 (− 0.070, 0.025)
*Females*						
T1	0.027 (− 0.060, 0.115)	0.027 (− 0.162, 0.217)	− 0.065 (− 0.128, − 0.001)	− 0.002 (− 0.077, 0.073)	− 0.221 (− 0.502, 0.059)	− 0.001 (− 0.074, 0.072)
T2	0.055 (− 0.010, 0.120)	− 0.109 (− 0.251, 0.033)	** − 0.054 (− 0.102, − 0.007)**	− 0.033 (− 0.089, 0.024)	0.079 (− 0.128, 0.286)	0.032 (− 0.023, 0.087)
T3	− 0.025 (− 0.107, 0.057)	− 0.021 (− 0.203, 0.162)	0.028 (− 0.031, 0.087)	0.028 (− 0.043, 0.098)	− 0.008 (− 0.271, 0.254)	** − 0.088 (− 0.159, − 0.017)**
*Males*						
T1	− 0.078 (− 0.162, 0.005)	**0.273 (0.081, 0.465)**	0.037 (− 0.034, 0.107)	0.056 (− 0.039, 0.151)	0.221 (− 0.063, 0.505)	0 (− 0.081, 0.081)
T2	− 0.030 (− 0.088, 0.028)	0.069 (− 0.070, 0.207)	0.009 (− 0.040, 0.059)	− 0.021 (− 0.087, 0.045)	0.173 (− 0.027, 0.374)	0.024 (− 0.032, 0.081)
T3	0.023 (− 0.042, 0.089)	− 0.088 (− 0.247, 0.072)	0.012 (− 0.043, 0.066)	− 0.027 (− 0.102, 0.049)	− 0.131 (− 0.358, 0.096)	0.025 (− 0.037, 0.088)

All estimates are reported in log-odds scale and rounded to the third decimal point, in parenthesis are the 95% bootstrap confidence intervals. *p*-values < 0.05 are highlighted in bold**.** T1: 1st trimester, T2: 2nd trimester, T3: 3rd trimester, nRBC: nucleated red blood cells

Results of sensitivity analyses are available in Additional file 1: Tables S2 and S3. We observed consistent results for compositional analysis and individual cell-type analysis, after excluding *N* = 17 women with preeclampsia or eclampsia. Specifically, we observed consistent results indicating a significant association between higher exposure to first trimester PM_2.5_ and the proportion of syncytiotrophoblast cells among male infants. Moreover, the first and second trimester PM_2.5_ exposures remained marginally significant in their overall effect on placenta cell-type composition among female infants. These findings reinforce the robustness of our results even after accounting for the exclusion of participants with severe pregnancy complications.

## Discussion

In this study, we investigated the impact of prenatal exposure to PM_2.5_ on placental cell types using two distinct analytical methods in a sample of 226 mother–infant dyads from the Northeastern US urban area. While we did not detect significant effects of PM_2.5_ on placental cell types or composition in the overall study sample, sex-specific associations were observed. Specifically, among male infants, exposure to PM_2.5_ during the first trimester was associated with a decrease in syncytiotrophoblast proportions and increase in trophoblast cell-type proportions. Among female infants, we observed associations between PM_2.5_ exposure during the second and third trimesters and decreases in nRBCs proportions and stromal cells proportions.

To our knowledge, this is the first study to demonstrate a link between gestational exposure to fine particulate air pollution and placental cell-type composition in a sexually dimorphic manner. Although previous investigations have not specifically evaluated this topic, our results are concordant with multiple studies reporting sexually dimorphic associations between air pollution exposure during pregnancy and molecular outcomes in placenta [7, 12, 40, 41] as well as early life developmental outcomes in children [14, 36]. It is known that the sex of the placenta and its cells can influence placental biology [16, 42]. Sex differences in placental gene expression and epigenetic marks have been detected throughout gestation, and these are not limited to sex chromosomes [16, 25, 43]. Our results also suggest that the critical windows of the effects of gestational PM_2.5_ exposure on placental cell-type composition are specific to infant sex and cell type. Such as, we found that first trimester exposure had effects on syncytiotrophoblast and trophoblast cells among male infants, while among females, second and third trimester air pollution exposure was associated with changes in nRBCs and stromal cells. Sex differences in air pollution exposure critical windows of exposure on a range of adverse child health outcomes have also been reported [36, 44, 45]. Our study contributes to the knowledge on the sex and time-varying association between PM_2.5_ and placental outcomes, further underscoring the importance of considering sex and windows of susceptibility in environmental epidemiology research.

Trophoblasts are pivotal placental cells that originate early in gestation, with two main subtypes: undifferentiated trophoblasts, more abundant in early gestation and differentiated syncytiotrophoblasts, which increase as pregnancy progresses. Trophoblasts are essential for placental invasion and vascular remodeling and syncytiotrophoblasts, which are in direct contact with maternal blood, coordinate maternal and fetal interactions [21, 46]. Syncytiotrophoblasts are the major epithelial cell in the placenta and play a key role in nutrient and chemical transfer between the fetus and mother. Our finding may have important implications in that regard. Our findings of decreased syncytiotrophoblasts and increased trophoblasts in male infants exposed to higher levels of fine particulate matter in the first trimester align with other lines of in vitro and animal research. In vitro studies have found that PM_2.5_ and wood smoke particle exposure can induce inflammatory and cytotoxic effects, leading to accumulation in first trimester trophoblast cell lines [47–50]. Additionally, a recent mouse study showed increased trophoblast proliferation in placental tissue harvested at day 19 gestation in response to intranasal preconception and gestational exposure to particulate matter [51].

Among female infants, our findings indicate that second and third trimester PM_2.5_ exposure was associated with changes in placental cell-type composition, particularly a decrease in the proportions of nRBCs and stromal cells. Mesenchymal cells are an important cellular component in the placenta that provide structural support for trophoblasts and as well as being linked to placental angiogenesis and modulation of immune cells [52–54]. Notably, studies conducted in mice have demonstrated a link between PM_2.5_ exposure and impaired placental vascularization and development [6], as well as reductions in the counts of red blood cells within the placental labyrinth zone [55], suggesting compromised oxygen and nutrient exchange in placenta. Similarly, in a mouse study introduced earlier, particulate matter exposure was linked to a significant decrease in the abundance of stromal cells [51].

To date, most human studies investigating the effects of air pollution on the placenta have been primarily focused on epi/genomic markers, predominantly gene expression and DNA methylation, in bulk tissue samples with limited exploration of effects at the cellular level [10]. The effects of air pollution on placental morphology and/or cellular composition remain largely unexplored. Few studies have examined associations between air pollutants and placental histopathology. For instance, a study on multiple household air pollutants, including PM_2.5_, found an association with increased risk of fetal vasculopathy in pathology examinations in a Tanzanian cohort of mother–infant pairs [56]. Similarly, another study on household air pollution exposure conducted in Nigeria, found associations between the use of firewood/kerosene and alterations in placenta pathology, specifically increased presence in Hofbauer cells, syncytial knots and chorionic vascular density, which are markers of chronic hypoxia in placentas [57]. However, investigations specifically focusing on the effects of ambient air pollution on placental cellular composition and detailed characterization of individual cell types have been scarce until now.

This study has several strengths. We leverage placental tissue DNA methylation data to evaluate the effects of PM_2.5_ exposure on placental cell-type composition. We employed two different analytical approaches. The study population is urban and diverse including a majority of black and Hispanic participants. Yet, this study is not without limitations. First, our analyses were not adjusted for multiple comparisons and statistical power may have limited our ability to detect some associations due to the relatively small sample size. Future research to replicate these findings should include a larger sample size. Second, the interpretation of our study findings is challenging due to the novelty of DNA methylation-inferred placental cell types as biomarkers. Changes in the abundance of placental cell types cannot be directly linked to specific pathophysiological processes, necessitating further investigation to elucidate the biological significance of these cell type changes. Additionally, it is important to recognize that placental cell types derived from a single chorionic villi tissue sample obtained at delivery may not fully represent overall changes in the larger placenta or effects seen in the earlier stages of gestation. Additionally, the reference-based deconvolution analyses in this work are limited to a small set of six common cell types present in the DNA methylation reference and may not capture changes in other less abundant cell types that exist in the placenta. Future studies should leverage single-cell technologies to build a more comprehensive placental reference DNA methylation panel that includes rarer cell types. Finally, DNA methylation biomarkers in heterogeneous tissues can be affected by cell-type composition, environmental exposures and disease processes [58]. Environmental exposures such as PM_2.5_ might cause inaccuracies in cell-type deconvolution, particularly if it influences methylation at cell-type specific CpG sites and if the exposure level is widely different than that of the reference population used to train the deconvolution algorithm.

## Conclusions

In summary, this is the first epidemiological study that examined associations between gestational PM_2.5_ exposure and placental cell-type composition inferred from epigenomic data. Our results reveal sex- and trimester-specific associations, demonstrating changes in the proportions of trophoblasts, nucleated red blood cells and stromal cells in response to PM_2.5_ exposure. These findings may inform future studies considering the sex-specific effects of prenatal air pollution exposure and their potential impacts on developmental outcomes in children.

### Supplementary Information

**Additional file1** File containing additional tables summarizing the results of the sensitivity analysis after excluding participants with eclampsia or preeclampsia.

## Data Availability

Data used in these analyses are available upon reasonable request in line with regulatory guidelines from the Director of the PRISM cohort study, Dr. Rosalind Wright. The code for compositional regression analyses is available at https://github.com/hasdk/compositional_regression.
