# Supplementary material for: DNA methylation profiles reveal sex-specific associations between gestational exposure to ambient air pollution and placenta cell-type composition in the PRISM cohort study
Source: Clin Epigenetics. 2023 Dec 1;15:188. doi: 10.1186/s13148-023-01601-x (PMC10693032; doi:10.1186/s13148-023-01601-x)
Supplement: Supplementary file 1 — Additional file1 File containing additional tables summarizing the results of the sensitivity analysis after excluding participants with eclampsia or preeclampsia. [file 13148_2023_1601_MOESM1_ESM.docx]

**DNA methylation profiles reveal sex-specific associations between prenatal ambient air pollution and placenta cell type composition in the PRISM cohort study**

**Supplemental tables**

**Table S1.** Median and interquartile range (IQR) of PM_2.5_ exposure levels in study participants.

|  | All (N=226) | Female (N=101) | Male (N=125) | *P*-value* |
| --- | --- | --- | --- | --- |
| PM_2.5_ exposures, median (IQR) | | | | |
| Pregnancy | 8.36 (1.22) | 8.41 (1.26) | 8.36 (1.22) | 0.35 |
| First trimester | 8.19 (1.81) | 8.31 (1.92) | 8.19 (1.81) | 0.35 |
| Second trimester | 8.27 (1.45) | 8.26 (1.51) | 8.27 (1.46) | 0.69 |
| Third trimester | 8.44 (2.08) | 8.65 (2.07) | 8.45 (2.12) | 0.23 |

******P*-value calculated using Brown-Mood median test

**Table S2.** Point estimates and 95% bootstrapped confidence intervals and *p*-values for the effects of PM2.5 estimated with compositional regression analysis. Preeclampsia and eclampsia (N=18) are excluded.

| **Effect of PM2.5** | **Syncytio-trophoblasts** | **Trophoblasts** | **Stromal** | **Endothelial** | **Hofbauer** | **nRBC** | ***P*-value*** |
| --- | --- | --- | --- | --- | --- | --- | --- |
| ***Overall*** |  |  |  |  |  |  |  |
| T1 | -0.012  (-0.034,  0.008) | 0.015  ( -0.001,  0.036) | -0.004  (-0.011,  0.002) | 0.002  (-0.003,  0.007) | 0  (-0.001,  0.002) | -0.001  (-0.003,  0.001) | 0.140 |
| T2 | 0.008  (-0.006,  0.022) | -0.005  ( -0.015  0.008) | -0.004  (-0.009,  0.002) | -0.002  (-0.007,  0.003) | 0.001  (-0.001,  0.003) | 0.001  (-0.001,  0.003) | 0.080 |
| T3 | 0.001  (-0.017,  0.017) | -0.005  (-0.018,  0.011) | 0.005  (-0.002,  0.011) | 0.001  (-0.005,  0.007) | -0.001  (-0.002,  0.001) | -0.001  (-0.003,  0.001) | 0.080 |
| ***Female*** |  |  |  |  |  |  |  |
| T1 | 0.006  (-0.027,  0.034) | 0.003  (-0.019,  0.031) | -0.007  (-0.017,  0.003) | 0  (-0.007,  0.007) | -0.001  (-0.001,  0.001) | -0.001  (-0.003,  0.002) | 0.178 |
| T2 | **0.020**  **(0.0001, 0.036)** | -0.012  (-0.025,  0.006) | -0.006  (-0.013,  0.003) | -0.004  (-0.011,  0.002) | 0  (-0.001,  0.003) | 0.001  (-0.001,  0.004) | **0.040** |
| T3 | -0.005  (-0.029,  0.018) | -0.002  (-0.022,  0.021) | 0.004  (-0.003,  0.012) | 0.005  (-0.002,  0.012) | 0  (-0.001,  0.002) | **-0.003**  **(-0.005,**  **-0.001)** | **0.040** |
| ***Male*** |  |  |  |  |  |  |  |
| T1 | **-0.037**  (**-0.068,**  **-0.009)** | **0.033**  **(0.006,**  **0.069)** | -0.001  (-0.010,  0.008) | 0.003  (-0.005,  0.011) | 0.003  (-0.002,  0.012) | -0.001  (-0.004,  0.002) | **0.049** |
| T2 | -0.007  (-0.029,  0.017) | 0.004  (-0.013,  0.021) | -0.001  (-0.009,  0.007) | 0.001  (-0.007,  0.008) | 0.002  (-0.001,  0.013) | 0.001  (-0.001,  0.003) | 0.210 |
| T3 | 0.011  (-0.019,  0.029) | -0.010  (-0.025,  0.010) | 0.004  (-0.005,  0.013) | -0.003  (-0.011,  0.006) | -0.002  (-0.003,  0.001) | 0.001  (-0.002,  0.003) | 0.062 |

In parenthesis are the 95% bootstrap confidence intervals. Significant results p<0.05 are highlighted in **bold**. * Overall effects on cell-type composition p-value calculated based on the median F-statistic across 1000 bootstrap samples. T1: 1st trimester, T2: 2nd trimester, T3: 3rd trimester, nRBC: nucleated red blood cells.

**Table S3.** Point estimates and 95% confidence intervals and P-values for the effects of PM2.5 estimated with Beta regression models. Preeclampsia and eclampsia (N=18) are excluded.

| **Effect of PM2.5** | **Syncytio-trophoblasts** | **Trophoblasts** | **Stromal** | **Endothelial** | **Hofbauer** | **nRBC** |
| --- | --- | --- | --- | --- | --- | --- |
| ***Overall*** |  |  |  |  |  |  |
| T1 | -0.014  (-0.076,  0.049) | 0.135  ( -0.007,  0.277) | -0.025  (-0.075,  0.024) | 0.030  (-0.032,  0.092) | -0.012  (-0.217,  0.194) | -0.003  (-0.060,  0.055) |
| T2 | 0.028  (-0.020,  0.076) | -0.054  ( -0.166  0.058) | -0.033  (-0.070,  0.005) | -0.021  (-0.069, 0.027) | 0.075  (-0.083,  0.232) | 0.022  (-0.023,  0.066) |
| T3 | -0.017  (-0.071,  0.038) | -0.031  (-0.159,  0.096) | 0.027  (-0.015,  0.070) | 0.004  (-0.050,  0.059) | -0.108  (-0.288,  0.072) | -0.038  (-0.089,  0.013) |
| ***Female*** |  |  |  |  |  |  |
| T1 | 0.028  (-0.060,  0.116) | 0.032  (-0.164,  0.228) | -0.065  (-0.130,  0.001) | -0.006  (-0.082,  0.071) | -0.212  (-0.501,  0.077) | -0.017  (-0.093,  0.060) |
| T2 | 0.069  (-0.001, 0.138) | -0.133  (-0.286,  0.021) | **-0.052**  **(-0.103,**  **-0.001)** | -0.049  (-0.110,  0.011) | 0.094  (-0.130,  0.319) | 0.023  (-0.037,  0.084) |
| T3 | -0.039  (-0.121,  0.043) | -0.010  (-0.196,  0.177) | 0.030  (-0.031,  0.090) | 0.043  (-0.028,  0.114) | -0.024  (-0.293,  0.244) | **-0.092**  **(-0.165,**  **-0.019)** |
| ***Male*** |  |  |  |  |  |  |
| T1 | -0.072  (-0.158,  0.015) | **0.279**  **(0.076,**  **0.482)** | 0.027  (-0.045,  0.100) | 0.063  (-0.033,  0.160) | 0.233  (-0.060,  0.526) | 0.004  (-0.081,  0.088) |
| T2 | -0.025  (-0.091,  0.041) | 0.036  (-0.123,  0.195) | -0.006  (-0.061,  0.050) | 0.010  (-0.063,  0.084) | 0.104  (-0.120,  0.328) | 0.023  (-0.041,  0.087) |
| T3 | 0.018  (-0.053,  0.089) | -0.085  (-0.259,  0.090) | 0.018  (-0.041,  0.077) | -0.035  (-0.115,  0.046) | -0.233  (-0.480,  0.013) | 0.005  (-0.064,  0.073) |

All estimates are reported in log-odds scale and rounded to the third decimal point, in parenthesis are the 95% bootstrap confidence intervals. *p*-values<0.05 are highlighted in **bold.** T1: 1^st^ trimester, T2: 2^nd^ trimester, T3: 3^rd^ trimester, nRBC: nucleated red blood cells.
